# Exploring assessor cognition as a source of score variability in a performance assessment of practice-based competencies

**DOI:** 10.1186/s12909-020-02077-6

**Published:** 2020-05-25

**Authors:** Mary Roduta Roberts, Megan Cook, Iris C. I. Chao

**Affiliations:** Department of Occupational Therapy, Faculty of Rehabilitation Medicine, University of Alberta, Edmonton, Alberta T6G 2G4 Canada

**Keywords:** Assessor cognition, Generalizability theory, Mixed method, Occupational therapy, OSCE, Performance assessment, Think aloud, Validity

## Abstract

**Background:**

A common feature of performance assessments is the use of human assessors to render judgements on student performance. From a measurement perspective, variability among assessors when assessing students may be viewed as a concern because it negatively impacts score reliability and validity. However, from a contextual perspective, variability among assessors is considered both meaningful and expected. A qualitative examination of assessor cognition when assessing student performance can assist in exploring what components are amenable to improvement through enhanced rater training, and the extent of variability when viewing assessors as contributing their individual expertise. Therefore, the purpose of this study was to explore assessor cognition as a source of score variability in a performance assessment of practice-based competencies.

**Method:**

A mixed-method sequential explanatory study design was used where findings from the qualitative strand assisted in the interpretation of results from the quantitative strand. Scores from one objective structured clinical examination (OSCE) were obtained for 95 occupational therapy students. Two Generalizability studies were conducted to examine the relative contribution of assessors as a source of score variability and to estimate the reliability of domain and holistic scores. Think-aloud interviews were conducted with eight participants assessing a subset of student performances from the OSCE in which they participated. Findings from the analysis of think-aloud data and consideration of assessors’ background characteristics were used to assist in the interpretation of variance component estimates involving assessors, and score reliability.

**Results:**

Results from two generalizability analyses indicated the highest-order interaction-error term involving assessors accounted for the second-highest proportion of variance, after student variation. Score reliability was higher in the holistic vs. analytic scoring framework. Verbal analysis of assessors' think-aloud interviews provided evidential support for the quantitative results.

**Conclusions:**

This study provides insight into the nature and extent of assessor variability during a performance assessment of practice-based competencies. Study findings are interpretable from the measurement and contextual perspectives on assessor cognition. An integrated understanding is important to elucidate the meaning underlying the numerical score because the defensibility of inferences made about students’ proficiencies rely on score quality, which in turn relies on expert judgements.

## Background

Performance assessments (e.g., Objective Structured Clinical Examinations; OSCEs) are one method for assessing practice-based competencies within a simulated context in a direct manner in health professions education [1]. Performance assessments usually require direct observation of student demonstrations. Judgements related to the quality of that performance are often operationalized as ratings against success criteria, typically specified within a rubric. Although performance assessments can provide standardized rating schemes for assessors to evaluate students’ competencies, the subjectivity of human judgements introduces the potential for inter-assessor score variability that negatively impacts on assessment quality [2].

Assessor cognition is a growing area of research to investigate how assessors’ cognitive processes affect decision making and assessment quality. Previous studies have identified different perspectives on assessor cognition by which to view the causes of assessor variability, including “assessor as trainable” (aligning with a measurement perspective) and “assessor as meaningfully idiosyncratic” (aligning with a contextual perspective) [3]. The measurement perspective views that assessors can be trained to comprehend and apply pre-specified criteria. If inconsistency of judgements emerges and results in inaccurate assessment outcomes, assessor training is essential to enhance consistency and to raise assessors’ awareness and application of the assessment criteria. The contextual perspective views that assessors are meaningfully distinctive, where different inferences and judgements among expert assessors can support a more holistic and context-dependent interpretation of a student’s performance.

The variability in assessors’ decision making could be considered problematic for measurement quality. The differing cognitive processes of assessors in formulating judgements may result in inaccurate assessment outcomes due to factors other than the intended measured construct. Therefore, an examination of assessor cognition can provide insights into its contribution to score variability, reliability, and validity. 

To examine assessors’ thinking processes in performance assessments, prior research has employed qualitative or mixed-method approaches. By using quantitative methods, researchers have compared scoring between assessors or between groups to ascertain the source of score variability [4]. By using qualitative methods, cognitive processes were explored by administering semi-structured interviews or utilizing a think-aloud approach [5, 6]. The think-aloud approach can be used to qualitatively examine assessors’ cognitions during the judgement and scoring task [7]. For example, Pool and colleagues asked assessors to evaluate a student mock portfolio while thinking aloud in order to reveal assessors’ thinking processes when making their judgements [6]. Crisp used the think-aloud method to understand assessors’ reading strategies, emotional and social impacts, and behaviors while scoring a school-based project work [5]. And Yeates et al. investigated the sources of variance in assessor ratings on performance assessments by applying the think-aloud protocol [8]. Integrating a measurement perspective with a qualitative examination of assessor variability can help us to better explore what components are amenable to improvement through enhanced assessor training, and the extent of variability in certain performance assessment contexts when viewing assessors contributing their expertise.

The purpose of this study was to explore assessor cognition as a source of variability in a performance assessment of practice-based competencies within an occupational therapy program. Quantitatively, assessor variability was estimated from observed ratings of performance. Qualitatively, assessor cognition as a source of variability was examined using think-aloud interviews. Together, these perspectives provide an integrated picture of the nature and variability of assessor cognition when rendering judgments on student performance.

## Method

A mixed-method sequential explanatory study design was used where findings from the qualitative strand assisted in the interpretation of results from the quantitative strand [9, 10]. Ethical protocols were approved and adhered to, as specified by the authors’ institution’s Research Ethics Board.

### Quantitative Strand

#### Data sample

Ninety-five students within a course-based master’s program in occupational therapy participated in an OSCE at the end of their first year, or two semesters, of coursework. OSCEs occur four times within the 26-month occupational therapy program, one at the end of each semester of coursework. The OSCE aimed to assess the practice-based competency domain of “Expert in Enabling Occupation”. Enacting the role of an expert in enabling occupation requires the student to demonstrate competencies such as clinical reasoning, professionalism, and effective communication. The OSCE entailed the student communicating a clinical summary of a client case to their supervisor. This scenario simulated an authentic task that occupational therapy students would experience when participating in case conferences with the healthcare team or debriefing client cases with their clinical supervisor. The clinical summary typically included a description of the client’s occupational profile, the client’s concerns related to participation in everyday activities, initial assessment findings, and proposed intervention strategies.

#### Instrument

A rubric (Appendix) was used to assess students within 6 domains. The rubric was provided to the students two weeks in advance of the OSCE so that they could become familiar with the domains and criteria for performance. The domains were, Professionalism, Communication, Theory, Models, and/or Frames of Reference, Knowledge of the Client, Clinical Reasoning, and Evidence-Based Practice. Sample criteria within domains included: “Manages time for responses and discussion in the time frame given, demonstrates active listening and appropriate non-verbal cues” (Communication domain) and “Demonstrates ability to fully link theory, models, and frames of reference to clinical practice” (Theory, Models, and Frames of Reference domain). Assessors provided analytic scores for each domain from 1 (Needs Work) to 7 (Distinguished). To generate the overall score, assessors were instructed to consider the interaction as a whole and to score the student out of 10.

#### Participants

Fourteen members from the occupational therapy department assessed the students during the OSCE. Thirteen assessors were female; one was male. All assessors held a practice-to-entry degree in occupational therapy representing varied practice backgrounds including mental health, neurological rehabilitation, occupational rehabilitation, pediatrics, and chronic pain, across the continuum of care. Ten were academic faculty with between 3 and 25+ years of experience in their role. Three assessors were clinical faculty with between 1 and 4 years in the role, and one assessor was a PhD student in their fifth year. On average, participants had participated in between two and three OSCEs in its current form. Assessors were paired by the OSCE coordinator; all but two taught within the first year of the program and were familiar with the students and curriculum completed at this stage in the program.

#### Procedure

Within a group setting, the assessors were provided with an orientation to the OSCE by the OSCE coordinator one week prior to the event. Participants were briefed on the objectives of the OSCE and the purpose of the present study. To facilitate assessors’ development of a shared understanding of expected performance at this stage in the program, assessors were presented the rubric and directed to consider the performance descriptors and distinguishing features between categories for each domain. During the orientation session, assessors were given an opportunity to discuss and ask questions to clarify the rubric content, exam procedures, and assessor expectations. Assessors were instructed to score each student along each of the domains. Then, considering their performance overall, assessors were instructed to assign a holistic score. Each student was assigned to a pair of assessors (from here on will be referred to as “raters” within the generalizability analyses), who assessed the student independently. Seven non-overlapping pairs of raters assessed between 8 and 14 students each. To keep the OSCE administration within the available time and resources, student assessments were conducted concurrently among the seven rater pairs. Due to this arrangement, it was not possible to use the same standardized patient across rater pairs. All rater pairs were instructed to score the student independently both at the domain level and holistic level. At the end of the encounter, students were provided with brief verbal feedback by the raters, typically focusing on one area that was done well and one suggestion for improvement. As per standard practice, for the purposes of review in the event of an appeal, all OSCE performances were video-recorded, saved on an external hard drive and stored in a secure location for later viewing.

#### Data analysis

Domain and holistic scores were summarized through descriptive statistics. We first calculated the mean scores and standard deviations within each assessor and domain combination, then within each domain collapsed across assessors. A Pearson correlation analysis was completed using JMetrik [11] to explore the associations between each of the domains and between domain and holistic scores for each assessor. Missing data were handled using listwise deletion.

Two Generalizability studies (i.e., G-studies) were conducted to examine sources of score variability and reliability of domain and holistic scores: 1) two-facet G-study with students fully crossed with domains and raters (i.e., *p x d x r*), and 2) one-facet G-study with students fully crossed with raters (i.e., *p x r*). Different groups of students were assessed by different raters (i.e., rater:student) resulting in a nested design and sparse data. The *rater approach* was used to generate variance component estimates for each rater pair using a publicly available SAS macro [12–14]. The weighted average of the variance components taken across the rater pairs was completed in Microsoft Excel.

### Qualitative Strand

#### Participants and data source

All 14 faculty members who participated as assessors in the OSCE were invited to participate in the think-aloud interviews. Eight faculty members gave consent to participation in the study. Of the eight, two were matched pairs from the original OSCE. Seven participants were female; one was male. Five were academic faculty with between 3 and 25 years of experience in that role. Three participants were clinical faculty with between 1 and 4 years in the role. On average, participants had participated in between two and three OSCEs in their current form.

OSCE videos were sampled based on the overall score to represent three levels of performance to ensure variability: low (60–70%), moderate (71–83%), and high (84–100%). Within one session, each participant reviewed two videos at each level of performance for a total of 2 X 3 = 6 videos. This set up simulated the pacing of the actual OSCE event where students were scheduled in sets of 5–6 students at a time. Assessors were not made aware whether they were viewing a low- or high-ranking student, nor did they have access to their original scores. Therefore, assessors rated the students in real-time without comparison.

#### Procedure

Using protocol analysis procedures, participants were individually interviewed and asked to think-aloud as they watched six video-recorded exams in which they participated from the original OSCE administration [7]. Participants verbalized their thinking, concurrently and retrospectively, during their assessment of the student using criteria specified within the original rubric. In total, 48 think-aloud interviews were audio-recorded and transcribed verbatim.

#### Data analysis

Think-aloud data were analyzed using verbal analysis to identify observed indicators of performance attended to and factors influencing the assessment of student performance [15]. Transcripts were segmented by “reasoning chain” as the level of analysis was congruent with the pattern and content of verbalizations during the think-aloud interviews [15]. Codes were developed in line with the purpose of facilitating explanation of the sources of variation identified through the generalizability analyses. Content was noted to examine assessors’ interaction with rubric components including alignment of their verbalizations with each of the domains and performance descriptions.

Both concurrent and retrospective verbal report data were analyzed. To begin, one author read through each transcript to familiarize themselves with the content and language used. This author developed an initial coding structure after open coding of six transcripts. Two authors independently applied the preliminary codes to a subset of six transcripts. Discrepancies in coding were discussed resulting in refinement of the initial code. The two authors used the revised code to recode the initial six transcripts. Due to high interrater agreement (> 95%), one author proceeded to independently code the remaining transcripts. New codes were developed for data that did not fit within the revised coding structure. After all transcripts were coded, a third coder independently coded a random sample of 12 (25%) transcripts to provide a check on consistency with discrepancies resolved through consensus. Guided by the research questions, patterns were sought within the data once coding was completed. Particular attention was paid to consistency across participants in their assessment and scoring of students by domain to examine assessor interactions. Verbalizations were then examined for common themes across participants.

### Integration of quantitative and qualitative strands

Ideally with measurement, variation in observed scores should be due primarily to true score variation between students on the assessed competency. However, our measurements are always imperfect to some degree, and there are likely other sources influencing observed score variation. Sources of variability other than the intended assessed competency would be considered a contribution to measurement error. Generalizability theory is a useful framework for unpacking measurement error into sources of variability that can be modeled and its influence on measurement quality assessed. In particular, generalizability theory allows us to examine the contribution of systematic and random sources of variability to observed score variation. Systematic sources of variability – such as raters/assessors and domains – are sources that are predicted to have a consistent effect on measurement. Random sources of variability – such as student mood or illness on the testing day – are ones that do not have a consistent effect on measurement. Good measurement endeavours to minimize measurement error in order to improve measurement quality.

In the context of this study using an observational, rater-based performance assessment, generalizability theory, supported by a correlation analysis, allowed us to examine the relative contribution of assessor and domain variation to the observed score. The mixed-method approach allowed us to examine assessor variability from the perspective of assessor cognition using think-aloud interviews. Variation in what aspects of performance were attended to, interpreted, and then scored by the assessors, contributes to assessor variability, and by extension holistic and domain score variability, as estimated using generalizability theory. Findings from the analysis of think-aloud data and consideration of assessors’ background characteristics were used to assist in the interpretation of estimates of assessor variability, assessor interactions, and reliability. Specifically, how does assessor cognition as a source of variability compare when captured statistically and qualitatively?

## Results

### Quantitative Strand

#### Distribution of ratings

Table 1 presents the mean scores within domain and assessor in the range of 4.15 to 6.43, and the average holistic scores among assessors in the range of 7.82 to 8.89. As the score of each domain and the holistic score ranged from 1 to 7 and 1 to 10, respectively, the mean scores indicate assessors assigned moderate to high scores in the OSCE. The standard deviations around the mean domain scores and holistic scores ranged from 0.39 to 1.82 and 0.34 to 1.07, suggesting variability in student performances as measured by the rubric and applied by the assessors.

**Table 1:** Mean ratings by domain and assessor

	**Assessor 1**	**Assessor 2**	**Assessor 3**	**Assessor 4**	**Assessor 5**	**Assessor 6**	**Assessor 7**
d1	5.14 (1.66)	5.14 (0.53)	5.50 (1.29)	5.23 (0.72)	5.82 (1.25)	5.23 (0.93)	5.36 (0.80)
d2	5.29 (1.44)	4.86 (1.23)	4.92 (1.71)	4.75 (1.22)	5.00 (0.77)	4.92 (0.95)	5.32 (1.14)
d3	5.29 (1.54)	5.14 (0.95)	5.36 (1.34)	5.46 (0.97)	5.80 (1.03)	4.67 (0.89)	4.82 (0.98)
d4	5.36 (1.39)	4.86 (0.95)	5.86 (0.95)	5.38 (1.04)	5.91 (1.30)	4.54 (0.88)	5.36 (0.81)
d5	5.29 (1.33)	5.07 (1.49)	5.71 (0.99)	5.46 (0.97)	5.00 (1.00)	4.62 (0.87)	5.00 (0.77)
d6	5.07 (1.82)	4.43 (1.60)	5.57 (0.85)	5.31 (0.85)	5.09 (0.94)	5.00 (1.34)	4.65 (1.11)
Holistic	8.31 (0.96)	7.89 (0.63)	8.89 (1.05)	7.96 (0.44)	7.64 (0.34)	8.12 (0.75)	7.96 (0.49)
	**Assessor 8**	**Assessor 9**	**Assessor 10**	**Assessor 11**	**Assessor 12**	**Assessor 13**	**Assessor 14**
d1	6.43 (0.76)	5.93 (1.32)	5.62 (0.87)	5.92 (1.04)	5.92 (1.04)	5.73 (0.90)	5.62 (1.04)
d2	6.08 (1.03)	6.14 (1.16)	5.38 (0.65)	4.92 (1.55)	5.92 (1.32)	5.91 (0.70)	5.08 (1.04)
d3	6.07 (0.99)	5.00 (1.46)	5.33 (0.65)	4.46 (1.05)	5.60 (0.97)	5.82 (0.87)	4.69 (1.18)
d4	6.00 (0.85)	5.14 (1.79)	5.62 (0.87)	4.92 (0.86)	5.62 (1.26)	5.82 (0.75)	4.15 (0.99)
d5	5.43 (1.01)	5.57 (1.45)	5.46 (0.78)	5.15 (1.21)	5.54 (1.39)	4.73 (0.65)	4.46 (0.88)
d6	5.54 (1.05)	4.92 (1.82)	5.17 (0.39)	4.46 (1.61)	4.83 (1.53)	5.27 (0.90)	4.77 (1.09)
Holistic	8.36 (0.93)	8.75 (1.07)	8.15 (0.47)	7.82 (0.71)	8.12 (0.85)	7.95 (0.39)	8.04 (0.85)

The score of each domain ranges from 1 to 7; the holistic score ranges from 1 to 10. Standard deviations are presented in parentheses

Note: d = domain; d1 = professionalism; d2 = communication; d3 = theory, models and/or frame of reference; d4 = knowledge of client; d5 = clinical reasoning; d6 = evidence-based practice

#### Correlations between domain scores and domain and holistic scores by assessor

Table 2 displays the correlations between domain scores for each assessor. Assessors 1–8 were the assessors participating in the think-aloud interviews. Correlations between domain scores for each assessor ranged from moderately negative to high (i.e., − 0.51 to 0.97). This variability in correlations between domains can be seen across assessors. For example, Assessor 1’s correlations between domains were generally moderate-high to high (i.e., 0.73 to 0.94). In comparison, Assessor 13’s correlations between domains were generally low to moderate (i.e., − 0.14 to 0.48).

**Table 2:** Pearson correlations between domains by assessor

	Assessor	d2	d3	d4	d5	d6	holistic
**d1**	1	0.92	0.86	0.91	0.89	0.89	0.96
	2	0.50	−0.04	0.04	0.37	0.46	0.51
	3	0.87	0.34	0.10	0.14	0.37	0.58
	4	0.47	0.45	0.31	0.66	0.68	0.53
	5	0.41	−0.20	0.10	0.28	0.10	0.15
	6	0.36	0.50	0.44	0.75	0.63	0.76
	7	0.61	0.65	0.38	0.65	0.64	0.73
	8	0.88	0.77	0.65	0.72	0.52	0.88
	9	0.70	0.59	0.52	0.58	0.70	0.77
	10	0.39	0.69	0.34	0.58	0.62	0.57
	11	0.36	−0.04	0.55	0.27	0.62	0.58
	12	0.25	0.79	0.35	0.39	0.11	0.68
	13	0.27	0.31	0.36	−0.14	0.10	0.29
	14	0.49	0.64	0.30	0.48	0.65	0.77
**d2**	1		0.79	0.94	0.88	0.82	0.88
	2		−0.51	−0.28	0.09	0.42	0.28
	3		0.44	0.35	0.28	0.60	0.79
	4		0.84	0.78	0.86	0.67	0.94
	5		0.53	0.62	0.69	0.68	0.83
	6		0.54	0.94	0.46	0.80	0.78
	7		0.57	0.18	0.34	0.69	0.91
	8		0.77	0.65	0.80	0.63	0.94
	9		0.40	0.50	0.45	0.33	0.71
	10		0.44	0.69	0.56	0.81	0.73
	11		0.33	0.37	0.85	0.51	0.69
	12		0.11	0.40	0.42	0.28	0.61
	13		−0.03	−0.03	−0.06	0.04	−0.02
	14		0.63	0.48	0.14	0.68	0.66
**d3**	1			0.77	0.79	0.73	0.87
	2			0.37	0.21	0.06	0.03
	3			0.72	0.78	0.75	0.78
	4			0.84	0.87	0.65	0.85
	5			0.46	−0.05	0.13	0.55
	6			0.55	0.82	0.59	0.66
	7			0.32	0.83	0.74	0.70
	8			0.62	0.60	0.65	0.82
	9			0.79	0.79	0.81	0.86
	10			0.26	0.44	0.37	0.79
	11			0.41	0.46	0.31	0.46
	12			0.63	0.61	0.53	0.83
	13			0.25	0.08	0.83	0.61
	14			0.69	0.55	0.84	0.84
**d4**	1				0.94	0.90	0.89
	2				0.23	0.45	0.36
	3				0.84	0.87	0.74
	4				0.71	0.32	0.66
	5				0.39	0.27	0.80
	6				0.46	0.77	0.81
	7				0.32	0.64	0.12
	8				0.64	0.35	0.68
	9				0.94	0.78	0.88
	10				0.51	0.59	0.55
	11				0.33	0.45	0.59
	12				0.97	0.73	0.71
	13				0.30	0.37	0.71
	14				0.68	0.50	0.44
**d5**	1					0.92	0.93
	2					0.53	0.71
	3					0.84	0.74
	4					0.82	0.92
	5					0.59	0.60
	6					0.67	0.75
	7					0.61	0.63
	8					0.73	0.82
	9					0.89	0.87
	10					0.81	0.73
	11					0.73	0.84
	12					0.72	0.71
	13					0.48	0.57
	14					0.29	0.48
**d6**	1						0.92
	2						0.70
	3						0.86
	4						0.75
	5						0.49
	6						0.78
	7						0.66
	8						0.69
	9						0.80
	10						0.69
	11						0.90
	12						0.65
	13						0.84
	14						0.91

Note: d = domain; d1 = professionalism; d2 = communication; d3 = theory, models and/or frame of reference; d4 = knowledge of client; d5 = clinical reasoning; d6 = evidence-based practice

*N* = 92 (listwise deletion)

The correlations between each domain and the holistic score ranged from very low to high (i.e., − 0.02 to 0.96). Most of the correlation coefficients between domain and holistic scores showed weak to moderate relationships, indicating variability between the associations of each domain to their holistic scores. For example, in comparison to the other domains, Assessor 2 had a low correlation between the domain of Theory, Models, and/or Frame of Reference and the holistic score (r = 0.03). In another example, Assessor 5 had a weak correlation between the domain of Professionalism and the holistic score (r = 0.15), but a high correlation between Communication and holistic score (r = 0.83), respectively. In contrast, Assessor 13 had negative correlations between Communication and with three other domains, and the holistic score (r = − 0.02 to − 0.06). Assessor 1 provided another point of comparison, where all domain scores were strongly correlated with the holistic score (r = 0.87 to 0.96). Examination of the raw data revealed that Assessor 1 gave the same score for every single domain for half of the students; this may have led to the high correlations between all domain and holistic scores.

#### Correlations between domain and holistic scores collapsed across assessors

Table 3 shows that the domains of Communication (r = 0.69), Clinical Reasoning (r = 0.72), and Evidence-Based Practice (r = 0.71) were most correlated with the holistic score, whereas the remaining three domains of Professionalism, Knowledge of the Client, and Theory, Models, and/or Frames of Reference were less correlated (r = 0.57 to 0.62). Overall, the average correlation between the domain and holistic scores was moderate (r = 0.66).

**Table 3:** Pearson correlations between domains and the holistic score

	d2	d3	d4	d5	d6	holistic
d1	0.59	0.45	0.40	0.44	0.52	0.62
d2		0.44	0.45	0.47	0.51	0.69
d3			0.64	0.54	0.59	0.62
d4				0.64	0.61	0.57
d5					0.68	0.72
d6						0.71

Note: d = domain; d1 = professionalism; d2 = communication; d3 = theory, models and/or frame of reference; d4 = knowledge of client; d5 = clinical reasoning; d6 = evidence-based practice

#### G-study analysis for analytic scores

Table 4 summarizes the weighted average of the seven variance components and the proportions of variance from the two-facet G-study. The percentages of variance associated with students, domain, and raters were 40, 3, and 3%, respectively. The percentage of variance associated with students, the object of measurement, should be high relative to variance attributable to domain and raters, as is the case here. The variance components for domain and raters were relatively small indicating that students performed somewhat differently across the six domains with regards to their assessment and that raters were fairly consistent in their rating behavior.

**Table 4:** Weighted average of variance components and proportions for student performance ratings from a two-facet fully crossed p x d x r design

*Source*	*df*	*SS*	*MS*	*Variance estimate*	*Proportion*
*p*	9.3	88.65	9.55	0.64	0.40
*d*	5.0	8.87	1.77	0.03	0.03
*r*	1.0	4.06	4.06	0.05	0.03
*p x d*	46.4	35.09	0.76	0.16	0.13
*p x r*	9.3	10.30	1.11	0.12	0.08
*d x r*	5.0	6.90	1.38	0.08	0.05
*p x d x r, e*	46.4	20.39	0.44	0.42	0.29

The percentage of variance for the two-way interactions involving raters ranged from 5% (*d x r*) to 8% (*p x r*). The relatively low percentages of variance associated with the two-way rater interactions suggests that there was some inconsistency between rater domain scores across students and rater scores of students across domains. After student variation, the next largest percentage of variance was associated with the 3-way interaction-error (*p x d x r, e)* term at 29%. This outcome suggests that the relative standing of students varied across domains and raters with potentially other systematic influences on the assessment of student competencies that have not yet been accounted for (e.g., unidentified facets, confounded effects). A generalizability coefficient of *G* = 0.80 and a phi coefficient of ϕ = 0.77 were obtained as measures of score reliability for relative and absolute decision-making, respectively.

#### G-study analysis for holistic scores

Table 5 presents the weighted average of the seven variance components and the proportions of variance from the one-facet G-study. The percentage of variance associated with students and raters were 81 and 7% with the object of measurement accounting for the largest proportion of variance. The two-way interaction, error term accounted for 13% of the total variance. This outcome suggests that differences between students varied to some extent by rater as well as by other unaccounted systematic influences. A generalizability coefficient of *G* = 0.93 and a phi coefficient of ϕ = 0.89 were obtained as measures of score reliability.

**Table 5:** Weighted average of variance components and proportions for student performance ratings from a one-facet fully crossed p x r design

*Source*	*df*	*SS*	*MS*	*Variance estimate*	*Proportion*
*p*	12.00	1315.64	105.77	49.35	0.81
*r*	1.00	41.71	41.71	2.86	0.07
*p x r, e*	12.00	88.29	7.07	7.07	0.13

### Qualitative Strand

#### Holistic scoring

We first examined verbalizations for similarities regarding performance indicators attended to. Similarities provided a source of evidence for consistency among the assessors’ thought processes.

#### Consistency of characteristics describing strong and weak performance

The verbalizations demonstrated that assessors were generally consistent with each other in identifying excellent and poor performance. Notably, the assessors used similar phrasing to describe overall strong and weak performances across their sample of students. Summarized across assessors, characteristics of an overall strong performance included:

- Appropriate non-verbal communication (e.g., eye contact, facial expression, and hand gesturing) was used effectively, congruent with what the student was saying, and not excessive to the point of distraction
- Professional appearance in attire and grooming
- Confident demeanour and management of emotions (e.g., anxiety) so that it did not interfere with performance
- Answering the question directly, did not evade or “talk-around” the question
- Answering the question reasonably quickly which was interpreted as the student “knows it”
- Responding in a concise yet organized manner only reporting relevant details
- Ability to spontaneously link across multiple content areas which was interpreted as a higher-level skill
- Responding flexibly to the demands of the interaction and could “think on their toes” without getting flustered or defensive

Characteristics of weaker performance were phrased opposite of those characterizing stronger performance. For example, a weaker student performance required cuing to make the connection between the client’s substance abuse and its impact on pain management, as opposed to, spontaneously making the connection between substance abuse and pain management which was indicative of stronger performance. Examination of the list of characteristics demonstrates what appears to be the collective importance of performance and presentation skills.

#### Variability in holistic scoring

We also examined how assessors formed an overall evaluation and numerical score. In particular, we examined verbalizations regarding the relative contribution of individual domains to the holistic score. No apparent pattern emerged across assessors. We interpreted this finding as evidence supporting assessor cognition as a source of variability contributing to the holistic score. For some assessors, it appeared that holistic scoring captured a global impression formed around the student’s performance as illustrated in the following excerpt.

So I would probably go around 83 percentage and that’s more based on just my overall sense of, like really wishing she would’ve been able to interconnect some of those really basic main components of the client’s presentation. (Assessor 3)

Assessor 3’s evaluation made reference to the feature of interconnecting basic main components of the client’s presentation. However, it is not clear which dimensions were being referenced in the verbalization, perhaps Knowledge of the Client and Clinical Reasoning. For some assessors, holistic scores were assigned with little elaboration. We interpreted the lack of verbalization as possibly an increased cognitive load demand to articulate and make explicit the process of assigning a numerical holistic score.

#### Analytic scoring

We examined verbalizations across assessors in the assessment and scoring of the domains noting patterns of consistency and divergence which may provide support for the results from the generalizability analysis.

#### Consistency with understanding of the domains

Examination of the verbalizations revealed that during the scoring task, assessors appeared to have consistent understandings of the domains at a broad level. However, it was also evident in the think-aloud interviews that although the domains were referenced generally, assessors did not consistently refer to the rubric performance descriptors as the OSCE unfolded. Explicit referencing of rubric performance descriptions occurred in less than 5% of the total verbalizations across participants. Assessors often verbalized their concurrent observations and inferences at a finer grain of description using their own words, in comparison to performance criteria described at a larger grain of description within the rubric. For example, assessors commonly took note of specific non-verbal behaviors such as eye contact, hand movements, and body posture:

Her hands are too busy and that would have been a comment on professionalism or communication. (Assessor 5)

Good professionalism. You know, good eye contact. (Assessor 6)

She has very good body posture and very clear communication. (Assessor 7)

Communication wise – well she used quite a lot of gestures and things but it’s okay because she’s not anxious too much. That kind of gestures is actually help engaging the conversation. (Assessor 1)

Non-verbal behaviors the assessors attended to generally aligned with the rubric dimensions of Professionalism and Communication. Although the assessors commented on specific observable behaviors, such as eye contact, these were not explicitly mentioned in the performance descriptors within the rubric. It appears that there may have been a shared or tacit understanding across assessors on what observable behaviors corresponded to effective communication.

#### Variable performance expectations

It appeared that assessors’ frames of reference (e.g., content expert, clinical preceptor) or educational perspective (e.g., curriculum, clinical practice) were variable and influenced their expectations during the assessment and scoring task. A priori expectations held by the assessors informed their comments on aspects of the student’s performance. For example, in the following excerpt, Assessor 1’s expectations appeared to be informed by their knowledge of the curriculum and how students should demonstrate this knowledge within the OSCE. In particular, Assessor 1 expected the student to consider the client’s needs holistically which is in keeping with the core principles of occupational therapy practice.

Theory wise and frame of reference, I would expect she [student] address a little bit more on maybe physical and social aspect and cognitive aspect as well. (Assessor 1)

As another example, Assessor 3’s expectations appeared to be informed from the dual perspectives of a clinician familiar with physical interventions and as an educator within the program. This working knowledge of clinical practice and students’ knowledge and skills at this stage in the curriculum then framed their expectations regarding the adequacy of clinical reasoning.

So she’s linking the vacuuming to the shoulder and that’s going to be difficult. I know they [the students] don’t have a lot of experience yet and that part of this could be a lack of experience in the level that they are at but then again it’s that clinical reasoning behind that. So right now, I’m missing a little bit of link between, how are you not causing pain in that shoulder? (Assessor 3)

For some assessors, expectations regarding the student’s responses were not directly linked to the rubric criteria, perhaps reflecting more personal expectations on how the student’s responses should unfold. These expectations could likely influence assessors’ evaluations potentially contributing a source of idiosyncratic variance in the observed scores. Assessor 3 illustrated this kind of expectation regarding any assumptions made by the student when designing a treatment plan. For Assessor 3, identifying and discussing assumptions provided a clearer window into the students’ clinical reasoning process:

So why are you making that assumption but also, what are some of the other assumptions that you could make and that you don’t think are relevant in this aspect … ? So to really give me some insight in why you’re thinking what you’re thinking, why are you making that assumption and not a different assumption? (Assessor 3)

As another example, the language used in one student’s response set up an expectation for Assessor 5 regarding communication of the client’s intervention plan to the clinical supervisor.

So I’ve had to give a more definitive description on what progression of a treatment is. I would’ve expected that to have been a little more spontaneous, in that we would start here and then we moved here and what types of things we would add or take away to test acquisition of strength and mobility or what, given that it is a wrist injury. (Assessor 5)

#### Variability in domain scoring

The salient observations made that would later inform scoring were not the same across all students for each assessor. We interpreted this finding as assessors reading into the rubric different things for different students. In this context, we had the rubric domains, assessor, and student coming together in an idiosyncratic way. To further explore this variability, we examined the verbalizations of two pairs of assessors, who were paired together during the original OSCE and also participated in think-aloud interviews, assessing the same student. The following examples demonstrated variability of thought processes among assessors while scoring the same student performance in the domains of Communication, Evidence-Based Practice, and Knowledge of Client.

Communication-wise, but I think she needs to be more concise in her communication, so she can get more information, more useful information, in there. (Assessor 2’s judgement on Student R)

Her communication is very clear and she's able to answer all questions. She has a very good body posture and very clear communication. (Assessor 7’s judgement on Student R)

Her evidence-based practice, she was able to give quite a bit of detail about the different things they looked at, but maybe not as much detail about what was the evidence about those specific components of the group program. (Assessor 2’s judgement on Student P)

Evidence-based priorities, she's good at this part. She can demonstrate very good knowledge about evidence based. (Assessor 7’s judgement on Student P)

She was giving a summary of the case and talking about the environment of the client, the social aspect of the client (was) pretty okay... Somehow, she’s able to explain like the client and show a little bit of understanding of the client. She talks a lot about the social aspect and the environment, which we think she has built an understanding of that. (Assessor 1’s judgement on Student S)

So she's giving a summary of her client. I felt that the summary was very weak... Knowledge with the client again didn't really know enough or go into enough depth on the client, so I'd give her intermediate for that.

(Assessor 8’s judgement on Student S)

These examples reflected that assessors had varying perspectives and interpretations even though they were assessing the same performance. While scoring the Communication domain, Assessor 2 considered the student’s delivery of their answers and concluded that there was room for improvement; whereas Assessor 7 considered the student’s body posture and ability to answer questions and concluded that the student demonstrated clear communication. In the second example, Assessor 2 thought that the student could provide more details regarding evidence; however, Assessor 7 thought that the student performed well in the domain of Evidence-Based Practice. In the last example, while judging the domain of Knowledge of Client, Assessor 1 focused on the student’s clarification on the social and environmental components of the client, and Assessor 8 seemed to reflect on the student’s overall knowledge of the client and concluded that the student did not dig deep to understand the client.

From the contextual perspective, differences in judgement between the paired assessors may represent legitimate differences instead of errors. Each assessor is an expert in the field of occupational therapy, and although the area of expertise may differ, each perspective is considered valid. Assessors’ prior knowledge and experience within the curriculum may have influenced their expectations, interpretation, and evaluation of performance against the rubric criteria for a specific domain. Given this, it seems plausible that the demonstration of complex or multi-faceted competencies could be interpreted in multiple, valid ways due to the interaction of the assessor and their expertise, the student, and the domain in which they are assessed.

### Integration of quantitative and qualitative strands

The pattern of correlations between domain and holistic scores varied across the assessors suggesting differential emphasis of the domains in the generation of the holistic score. Assessor verbalizations and assessor backgrounds together provided some explanation of the correlation results. For example, Assessor 2 had a low correlation between the domain of Theory, Models, and/or Frame of Reference and the holistic score (r = 0.03). Assessor 2, who was early in their role as clinical faculty, verbalized not feeling confident in their scores within the domain of Theory, Models, and/or Frame of Reference. Assessor 2 did not teach the course on theory, models, and frames of reference within the curriculum which may explain the low correlation between this domain with the holistic score.

Interestingly, the most experienced instructors (25+ years) in this study (i.e., Assessors 5, 6, and 14) demonstrated variable scoring patterns among each other. For example, Assessor 5 had a weak correlation between the domain of Professionalism (r = 0.15) and the holistic score, but a high correlation between Communication and the holistic score (r = 0.83). In a comment during the think-aloud interview, Assessor 5 remarked that they had their own “perception” for assessing students, comparing their judgement of one student against the rest, and with a large proportion of comments related to non-verbal communication and verbal explanations. This finding was also highlighted in a previous study by Govaerts et al., where they reported that experienced assessors had more distinctive judging schemas compared to novice assessors during a performance assessment. They concluded that experienced assessors were significantly triggered by task-specific cues linked to prior experience when judging performance [16].

The generalizability analysis results within the holistic scoring framework showed a high proportion of variance attributable to student performance (81%) and a relatively lower proportion of variance attributable to rater (7%). The findings from the think-aloud interviews support the generalizability analysis. Findings from the think-aloud interviews showed that assessors were generally consistent in discriminating between good and weaker performances with agreement on observable indicators of performance. This relative consistency across assessors is reflected in the lower variability components attributable to raters. However, differences in how holistic scores were assigned were also noted in assessor verbalizations and aligned with the results from the correlation analysis supporting the variance component estimate attributable to the interaction-error term at 12%. The contributions of the individual domains to the overall holistic score differed across assessors or assessors took an impressionistic approach in assigning the holistic score. Taken together, findings from the think-aloud interviews support the high reliability value reported with a generalizability coefficient of *G* = 0.93 and a phi coefficient of ϕ = 0.89 obtained, respectively.

The picture for analytic scoring is more complicated. Student variation accounted for the largest proportion of variance at 40% and rater variation (collapsed across domains and students) was 4%. Similar to the findings of holistic scoring, the qualitative findings support some level of consistency across assessors in the general understanding of the domains with an apparently shared understanding of performance indicating effective performance. The second-largest contributor of variance after student performance was attributed to the highest order interaction-error term (i.e., *p x d x r, e)* at 29%. The presence of this sizeable interaction term indicates that the relative ranking of student performance by scores was dependent to some degree on the domain in which they were assessed and the assessor involved. The variability among assessors’ performance expectations likely influenced the observations and interpretations made during the assessment task facilitating potentially idiosyncratic application of the scoring rubric across students. This interaction between student, assessor, and rubric application is captured in the presence of the non-negligible interaction variance components (i.e., highest-order interaction/error term) from the generalizability analysis. Taken together, findings from the analysis of assessors’ verbalizations support the reported reliability values for analytic scoring as lower in comparison to holistic scoring with a generalizability coefficient of *G* = 0.80 and a phi coefficient of ϕ = 0.77.

## Discussion

The purpose of this study was to examine assessor cognition as a source of score variability in a competency-based performance assessment within an occupational therapy program. We utilized a mixed-method design where results from a generalizability analysis were explored further qualitatively using think-aloud interviews. Generalizability analyses were completed to examine sources of variability and their relative contribution to holistic and domain score variability. Results from the generalizability analyses indicated that the largest source of variability was attributable to differences between student performances which, from a measurement perspective, is a desirable outcome. The results also indicated contributions to score variability that were attributable to interactions involving assessors, such as the highest order-error interaction term.

Verbal analysis of assessors’ cognitions provided insight into the quantitative results, specifically the reliability estimates obtained and explanation for the assessor (rater) interaction terms. For holistic scoring, assessors as a group were generally consistent in their characterizations of stronger and weaker performances and their ability to distinguish between the two. The generation of the holistic score varied between assessors with differential emphasis of the domains to the holistic score, as supported by the correlation results, or the use of a global, impressionistic approach. These findings supported the relatively high reliability of holistic scores with the largest proportion of variance attributable to student differences on the assessed competency. In comparison to holistic scores, the reliability for analytic scores was lower. Student differences accounted for the largest proportion of variance but was then followed by the highest-order interaction-error term (i.e., *p x r x d, e*). Assessor verbalizations demonstrated general understanding of the domains despite rarely referencing the rubric criteria explicitly in their assessment and scoring of performance. Additionally, assessors had variable performance expectations, influenced by their frame of reference or educational perspective. Variability was also noted in analytic scoring, where verbalizations of assessor pairs viewing the same student performance resulted in different judgements and assigned scores. Taken together, the qualitative findings supported the non-negligible variance component attributable to the highest-order interaction-error term, suggesting that despite participation in assessor training, variability remains in assessors’ cognition during the assessment and scoring of student performance.

The results of this study are consistent with previous studies examining assessor cognition. In a similar study design, Naumann et al. [4] examined factors contributing to examiner judgement using semi-structured interviews and generalizability analysis for an OSCE in exercise physiology. The pattern of results obtained by Naumann et al. [4] and the current study are the same with the largest proportion of variance in both holistic and domain scoring attributable to student performance. Naumann et al. [4] focused discussion of their study results around variability noted in the thought processes across examiners despite examiners (as a main effect) having minimal impact on variance. The current study adds to Naumann et al.’s [4] study with a focused examination on assessor interaction terms, specifically the highest-order interaction term in the analytic scoring framework, and estimation of reliability. Examination of the interactions is important to understand the extent and contribution of idiosyncratic variability in reported scores. This examination was enabled through analysis of concurrent and retrospective think-aloud interviews, in contrast to self-reported explanations, which reveal the cognitive processes used during assessment in real time.

The use of concurrent and retrospective think-aloud interviews to investigate assessor cognition was also used in a study by Yeates et al. [8] investigating the mechanisms that give rise to variability in scores. These mechanisms included differential salience, criterion uncertainty, and information integration. We believe that these three mechanisms were also at play in our study. Differential salience was observed in assessors’ verbalizations when they attended to different aspects of performance to differing degrees across students in analytic scoring. Criterion uncertainty was present in the current study with assessors’ variable frames of references informing performance expectations against which to judge performance even with the availability of the scoring rubric. Last, for information integration, assessors largely verbalized their thoughts in their own language at a finer grain of description when compared to the rubric performance descriptors. Similar to Yeates et al., assessors in the current study rarely referenced the rubric descriptors directly in their judgements of performance.

The findings of this study are interpretable from the measurement and contextual perspectives on assessor cognition [3]. Examining assessors’ cognition as they engage in the assessment and scoring of student performance in the OSCE provides a window into how assessor variability arises. Some assessor variability is expected given the OSCE requires human observation, interpretation, and scoring of performance. However, the magnitude of variance components involving assessor interactions, especially that of the highest order-error interaction term, warrant closer attention. A strategy to improve measurement quality is to address systematic sources of variability affecting observed scores. In the context of this study, assessors as a source of systematic variation presents as an area of focus for enhancing consistency. The measurement perspective views assessors as trainable “information processors” matching indicators of observed performance to criteria [17]. In this view, variability within analytic scoring could be addressed through enhanced rater training with the goal to improve scoring consistency, and subsequently, score reliability.

Interactions involving assessors may suggest potential issues with fairness, where a student’s score may be dependent on the assessor observing them and the domain in which they are being assessed. The verbalizations indicated assessors approached the assessment task with variable frames of reference or perspectives which influenced expectations for performance. It may be worthwhile to examine the current approach to assessor orientation and implement procedures where assessors discuss, or are shown, what student performance “looks like” with regards to each of the domains as described by the performance descriptors. This approach is aligned with the “frame of reference” rater training as described by Holmboe [18].

The contextual perspective on assessor cognition is where assessors are viewed as experts where differences represent legitimate and valid judgments [3]. In this view, variability of analytic scoring with the presence of interactions is expected. The results of this study show that even with the presence of assessor variability, reliability estimates were high with generalizability coefficients at 0.80 or higher for both holistic and analytic scores. These values suggest that perhaps there is some underlying consistency or shared understanding of student performance between the assessors for this particular OSCE. From a contextual perspective, these assessors may form a community of practice interested and involved in performance assessment of students. Through participation in a community of practice, assessors may continue to develop assessment skills and expertise further facilitating a shared understanding of students at this particular level.

Notably, in the context of the occupational therapy program within which this study is situated, different perspectives among assessors were seen positively by some. Occupational therapy models of practice embrace a contextual perspective. A client’s lived experience of health and participation results from a unique interaction between themselves, their everyday activities, and the environment in which they function. Tensions may arise with occupational therapy faculty as assessors, espousing a situated worldview and engaging in forms of assessment that prioritize standardization and reduced measurement error. Further exploration of assessment beliefs and faculty attitudes toward assessment would contribute to an understanding of acceptability and use of performance assessment in occupational therapy education.

### Limitations

A limitation of this study is the recruitment of eight assessors from the original 14 to participate in the think-aloud interviews. Additionally, the procedures for the think-aloud interviews simulated the conditions of the original OSCE where assessors viewed interactions in which they participated. Due to administration and resource considerations for the OSCE, this resulted in assessor pairs viewing a different standardized patient for their assigned group of students. Therefore, the results of this study are limited to the conclusions drawn from the eight assessors under realistic conditions. Moreover, the institutional background or type of program in which the assessor was educated and its impact on assessment was not examined. The study results may not generalize to OSCEs in other professional programs. However, professional programs might find the results useful for understanding assessors’ thinking and its influence on the scoring of performance against criteria within a rubric in real-time.

## Conclusion

This study contributes to health professions education by providing insight into the nature and extent of variability among assessors in performance assessment from two perspectives. In particular, this study investigated assessors’ cognition, though the use of think-aloud interviews, as a contributing source of variation to observed score variation within the context of an OSCE within occupational therapy education. An integrated understanding is important to elucidate the meaning underlying the numerical score and because the defensibility of inferences made about students’ proficiencies rely on score quality, which in turn relies on expert judgements. This study contributes to scholarly discourse on seemingly incompatible perspectives on assessor variability – as error, consistent with the measurement perspective, or as expected, consistent with the contextual perspective. In the context of occupational therapy education, the suggested next stage of inquiry is to explore relationships between assessors’ own professional values and their beliefs and attitudes towards performance assessment, particularly the acceptability and use of standardized forms.

## Data Availability

The datasets used and/or analyzed during the current study are available from the corresponding author on reasonable request.

## Appendix

**Table 6:** Mark the square that best represents the student’s performance

	Needs work	Intermediate	Proficient	Distinguished
**Professionalism**	Not presenting self professionally (attire, grooming) for interview.	Does not use appropriate professional language during interview.	Does not introduce self but otherwise conducts self professionally.	Introduces self clearly as sole occupational therapist, conveys a sense of self-confidence during interview.
**Communication**	Unable to build rapport during interview	Uses appropriate communication style to interact but needs to be more concise/organized in relaying information or lacks active listening skills.	Uses appropriate communication style to interact but some improvements could be made in relaying information non-verbal cues and/or evidence of active listening.	Manages time for responses, discussion in timeframe given, demonstrates appropriate non-verbal cues, demonstrates active listening
**Theory, models and/or frame of reference**	Unable to provide theory, model or FOR to apply to case study.	Limited ability to link theory, models or FORs to case study.	Partially able to link theory, models and FOR to clinical practice	Demonstrates ability to fully link theory, models and FOR to clinical practice
**Knowledge of client**	Basic knowledge of the client	Able to elaborate on the client however, difficulty linking the different components	Able to elaborate on the client linking some of the different components	In-depth understanding of client. Holistic, comprehensive of the different aspects
**Clinical reasoning**	Unable to provide clinical reasoning to support assessment findings, interventions and goals	Demonstrates incomplete clinical reasoning paired with explanation of client assessment findings, interventions and goals	Demonstrates adequate clinical reasoning paired with explanation of client assessment findings, interventions and goals	Demonstrates robust clinical reasoning paired with explanation of client assessment findings, interventions and goals
**Evidence- based practice**	Unable to articulate need for evidence-based practice	Demonstrates knowledge of evidence-based practice but unable to apply approach to clinical decisions for client	Demonstrates limited knowledge of evidence-based practice related to clinical decisions for client	Demonstrates robust knowledge of evidence-based practice related to clinical decisions for client

**Considering the whole interview, how would you rate the student’s performance?**

**______/10**

## Abbreviations

OSCE: Objective Structured Clinical Examination
G-Study: Generalizability Study
